# Bleomycin Sclerotherapy for Severe Symptomatic and Persistent Pelvic Lymphocele

**DOI:** 10.1155/2014/624803

**Published:** 2014-07-06

**Authors:** Ana Sofia Fernandes, Antónia Costa, Raquel Mota, Vera Paiva

**Affiliations:** ^1^Department of Obstetrics and Gynaecology, São João Hospital Center, Portugal; ^2^Medicine Faculty of Porto University, Portugal

## Abstract

*Background.* Pelvic lymphoceles are frequently described as a complication of pelvic lymphadenectomy performed for surgical staging of gynaecologic malignancies. *Case Report.* A 72-year-old woman presented with severe symptomatic and refractory lymphocele associated with persistent lower limb lymphedema and recurrent erysipelas. After four CT fluoroscopy scan guided percutaneous catheter drainages, the lymphocele complicated with infection finally resolved with two sessions of bleomycin sclerotherapy. *Conclusion.* Symptomatic persistent lymphoceles require treatment and nowadays the first option is interventional radiologic procedures. Bleomycin is a safe and effective sclerosing agent and therefore should be regarded as a first-line treatment choice.

## 1. Introduction

Lymphocele was first reported in 1950 by Kobayashi and Inoue [1] and it is defined as a lymph filled extraperitoneal collection with a fibromembranous wall devoid of any epithelial lining [2–4]. Therefore, lymphocyst terminology is not appropriate.

The etiopathogenesis is based on the disruption of the lymphatic vessels, which occur for traumatic or surgical reasons. Lymphoceles mainly develop after extraperitoneal surgery and much less frequently after intraperitoneal procedures, because the peritoneum plays a major role in the lymph absorption. The specific characteristics of lymph (namely, the flow tendency after injury due to its reduced clotting factors concentration and the absence of platelets) and of the lymph vessels (namely, the absence of smooth muscle in the vessel wall precluding vasoconstriction) explain the appearance of lymphoceles. These occur mainly after renal transplantation and urogynecologic malignancy related lymphadenectomy. Lymphocele formation after vascular and spinal surgery has been reported less commonly [5–9]. The lymphatic vessels can remain patent up to 48 hours after injury [10]. Lymphoceles generally develop a few weeks after retroperitoneal surgical procedures; however, late occurrences up to two years have been described [11].

The exact incidence of pelvic lymphoceles is not established; it has a wide variation depending on surgical approach, screening and diagnosis methods, and symptomatology. At pelvic lymphadenectomy for gynaecologic malignancies one can find reports of incidence that range between 18% [2] and 44% [12]. Considering only symptomatic postoperative lymphoceles, Achouri et al. reported an incidence of 34,5% [13]. In the particular case of symptomatic lymphoceles after complete cytoreductive surgery and para-aortic and pelvic lymphadenectomy for ovarian cancer, Gauthier et al. describe an incidence of 28% [14].

When asymptomatic, as most of the cases, spontaneous regression is the natural history [2, 12]. On the other hand morbidity has been described, such as infection and compression of adjacent structures (ureters with hydronephrosis; rectosigmoid with constipation; pelvic nerves with leg/back pain and urinary frequency; and vessels with genital/lower limb edema and thrombosis/embolism). Complications can emerge, namely, chylous ascites and lymphatic fistula [3, 15, 16].

In the presence of a severe symptomatic pelvic lymphocele, treatment must be offered not only to relieve symptoms and control the functional compromise of adjacent vital structure, but also to prevent a delay of the underlying disease treatment [16–18].

There are several treatment modalities. Nowadays, the first option is interventional radiology procedures (simple aspiration, image guided percutaneous catheter drainage with or without sclerotherapy), leaving surgical treatment for second-line therapeutic [4].

The authors report a case of a severe, symptomatic and refractory pelvic lymphocele successfully treated with bleomycin sclerotherapy.

## 2. Case Report

A 72-year-old woman was referred to our outpatient clinic for an abdominopelvic mass clinically suggestive of ovarian carcinoma. The patient relevant medical and surgical history was obesity (BMI 34.8 kg/m^2^), essential arterial hypertension, diabetes mellitus, dyslipidaemia, and iatrogenic hypothyroidism (total thyroidectomy for a benign condition).

Complete surgical ovarian cancer staging was performed and included total hysterectomy, bilateral salpingo-oophorectomy, pelvic and para-aortic lymphadenectomy, infracolic omentectomy, apendicectomy, and multiple peritoneal biopsies. Two continuous suction drains were placed at the end of the procedure in both iliac fossae, which were removed on the fourth postoperative day. For systemic thromboprophylaxis reasons she maintained low molecular weight heparin during the 30 days after surgery. Recovery was uneventful with discharge at the fifth postoperatory day.

The patient was evaluated on the 13th postoperative day. Definitive diagnosis was grade 2 ovarian mucinous adenocarcinoma, right pelvic ganglia 0/12, left pelvic ganglia 0/27, para-aortic ganglia 0/29, and FIGO surgical staging IAR0. She complained of pain and swelling of the left lower limb. On physical examination she presented left lymphedema and bilateral erysipela (more pronounced on the left). The abdominopelvic ultrasound revealed a bilateral pelvic lymphocele, the left with 109∗56 mm, and the right with 45∗21 mm. Bilateral lower limb venous* Doppler* excluded deep venous thrombosis.

**Figure 1:**
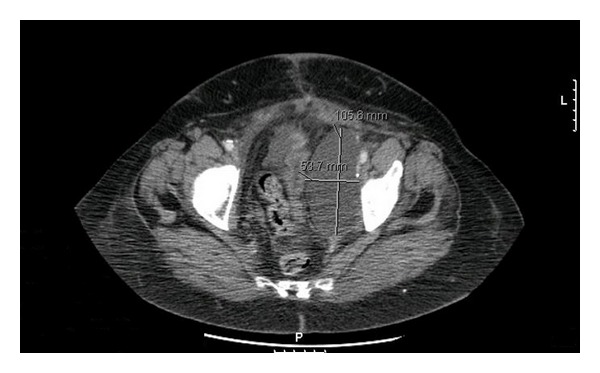
Pelvic lymphocele recurrence.

After six days of unsuccessful empirical antibiotherapy with flucloxacillin for the erysipelas, she was readmitted. A CT fluoroscopy scan guided percutaneous catheter drainage was performed. Externalization of a clear, slightly yellow-tinged, benign, and sterile fluid was achieved with trocar technique with a 7F Huisman catheter. There was a progressive improvement of the lymphedema and erysipelas, and the drain was removed six days after (last day output 200 mL/24 h with previous medium output/24 h of 850 mL), with ultrasound confirmation of left lymphocele shrinkage with 51∗40 mm; however, six days later she complained of excruciating left inguinal and lower left sided back pain. Imagiologic evaluation by ultrasound and CT pelvic scan revealed recurrence of left lymphocele with 106∗54 mm, impairing venous drainage of the inferior limb (Figure 1). Fifteen days after the first drainage, another CT fluoroscopy scan guided percutaneous complete drainage was performed (200 mL), using Seldinger technique with an 8F pigtail catheter, and was removed four days later (last day output 150 mL/24 h with previous medium output/24 h of 550 mL). Twenty-two days after the admission she was discharged with no pain and lymphedema improvement.

Two months after this discharge the patient was readmitted, complaining of pain and swelling of the left inferior limb, anorexia, and vomiting. Abdominopelvic ultrasound showed aggravated left lymphocele with 190∗90 mm, compressing the homolateral iliac vessels and ureter with left limb iliac-femoral-popliteal thrombosis. Analytic parameters were compatible with infected pelvic lymphocele and decompensated diabetes, and therefore she was admitted at the intensive care unit. Under low molecular weight heparin and antibiotic therapy (piperacillin/tazobactam) another CT fluoroscopy scan guided percutaneous catheter drainage was performed on the 7th day. Nearly complete aspiration of lymphocele was performed, using trocar technique with a 12F pigtail catheter. There was isolation of ampicillin resistant* Klebsiella pneumonia*. The pigtail catheter was removed 12 days after, and transition to endovenous cefazolin was performed in order to treat cellulites in the spot of the catheter and erysipelas. Fifteen days after and due to persistent and high output (total of 3100 mL in 4 days) pelvic lymphocele, fluoroscopy scan guided percutaneous catheter (pigtail) drainage was performed. Later, the empty cavity of the lymphocele was filled with contrast medium in order to exclude fistulous communications, which preclude sclerotherapy. 60 000 UI of bleomycin diluted in 50 mL saline was instilled into the lymphocele cavity with occlusion of the catheter, which was left in situ. During this time the patient was mobilized in order to achieve penetrance of the sclerosing agent in all the loci. After 60 minutes of patient mobilization, all the amount of the sclerosing agent was sucked by a syringe from the cavity. The procedure was well tolerated without any complication and was repeated seven days after. Pigtail catheter was removed one week later, after an abdominopelvic CT scan showed reduction of left lymphocele with 71∗28∗55 mm. She was discharged the next day.

The patient was submitted to a specific programme of physical rehabilitation to improve the motor dysfunction associated with the inferior limb lymphedema.

A control abdominopelvic CT scan performed one month after the last hospital discharge did not revealed any pelvic collection.

Three years after the surgery the patient remains in oncologic remission with improvement of the left inferior member lymphedema and no imagiologic relapse of the pelvic lymphocele.

## 3. Discussion

Although divergent results can be found in the literature, many factors are believed to contribute to pelvic lymphocele formation. It may be associated with heparin (some studies showing that the administration near the abdominopelvic region is prone to the occurrence of lymphocele) [19], diuretics, steroids, high body mass index, and surgical technique (extent of lymphadenectomy with the number of lymph nodes removed and the presence of metastases to the lymph nodes) [2, 18, 20–23].

Despite the absence of clear consensus on the risk factors, some surgical measurements may have a potential impact at decreasing the incidence of lymphoceles, such as prefer the laparoscopic approach, ligate the lymph vessels, avoid peritoneal juxtaposing at the end of the procedure [16], and preclude suction drainage tube placement [24] and, when feasible, omentoplasty should be encouraged [16].

In this case, several factors may have contributed to the pelvic lymphocele formation: the high body mass index, the extent of lymphadenectomy, the administration of heparin in the thigh and postoperative suction drainage. In our department, we perform thromboembolic prophylaxis with low-dose subcutaneous heparin to all oncologic patients who are submitted to pelvic surgery. Since the risk of deep vein thrombosis and fatal pulmonary embolism is very high, it justifies its widespread use and overweights the lymphocele risk. In the past, following radical pelvic and lomboaortic lymphadenectomy, we routinely performed surgical peritoneal juxtaposition and maintained retroperitoneal suction drainage placed at both the iliac fossae for about four days. Actually we still perform thromboprophylaxis during the perioperative period but no longer do the surgical peritoneal approximation or the suction drainage.

Even though almost complete evacuation of the cavity was achieved in each percutaneous drainage, an essential condition to avoid bacterial infection, the repetitive drainages with long term catheter placement and the poor glycaemic control were probably at the basis of the infection. As it is well known, hyperglycaemia in diabetes mellitus is a condition associated with high infection risk.

The treatment modalities describe the surgical approach and percutaneous techniques through interventional radiology (Table 1). The gold standard was open surgery by performing internal marsupialization with drainage into the peritoneal cavity internally added with omentoplasty. External drainage technique [25], also described, has major drawbacks as long hospitalization, relative high risk of infection, and a high recurrence rate (up to 25%) [3]. Thereby it is no longer a first option. The laparoscopic approach, first described in 1994 by McCullough et al. [26], has a success rate of 95% in the more recent series [27, 28] and nowadays it is the surgical approach of excellence in the literature. The benefits are inherent of every laparoscopic procedure.

The minimal invasive therapeutic options include simple aspiration, simple catheter drainage, and combined catheter drainage with transcatheter sclerotherapy. Simple aspiration should be performed only in small lymphoceles, because of the high risk of recurrence (80–90%) and infection (25%) [3, 29]. Percutaneous catheter drainage has a variable success rate of 70% to 100% [30–34]. The option of adding sclerotherapy has comparable success rates to surgery. Regarding the sclerotherapy, there is no consensus on the optimal sclerosing agent: povidone-iodine, sodium tetradecyl sulphate, sodium azetroate, sodium mortuate, talk, fibrin glue, ethanol, ampicilina, tetracycline, doxycycline, and bleomycin. These act by irritation of lymphocele's walls, inducing local inflammation and fibrosis of the lymphatic channels, obliterating the lymphatic leak. They can be used as a primary treatment or for persistent lymphoceles. Since there is no robust scientific evidence comparing the effectiveness and efficiency of the individual sclerosing substances (success rates range between 62 and 100% and recurrence rates between 3 and 38%), choosing one over another depends on personal preferences and experience [3, 4, 34–50]. The comparable success rate to the surgical approach has shifted the first-line treatment option to this minimal invasive sclerotherapy. This procedure can be performed on an outpatient basis, with no antibioprophylaxis needed in immunocompetent patients, and is executed under conscious sedation. Ultrasound and CT or MR imaging are useful to diagnose and characterize the lymphocele in respect to location, dimension, and relationship to adjacent structures. But cystograms are ideal for evaluation of the extension of the lymphoceles, to differentiate side to side from juxtaposed lymphoceles from a septate lymphocele and during the intervention it is essential to exclude extravasation outside the cavity of the lymphocele, which is the contraindication to do the sclerotherapy.

Bleomycin is a cytotoxic agent used systemically for the treatment of some malignant neoplasia. Topical use allows the formation of adhesions, making an excellent agent for sclerosis. There are some accumulated experiences using bleomycin for sclerosis of malignant pleural effusions [51, 52] and treatment of lymphangioma [53, 54]. It is not as frequently reported as other agents for lymphoceles sclerotherapy. After the first case of Khorram and Stern of a resistant inguinal lymphocele treated with bleomycin [55], Kerlan et al. reported successful use of this sclerosing agent in four patients, three resistant to other sclerosing agents. They stated that bleomycin should not be considered the agent of choice for sclerosis of postoperative lymphoceles, and it should be reserved for refractory lymphoceles to other sclerosing agents [50]. The reason for this advice is that it is associated with potential side effects, like fever, nausea, vomiting, stomatitis, skin rash, chest pain among others, and cost. The exact incidence of these collateral effects with lymphocele esclerotherapy has not been reported but is probably less compared with intravenous chemotherapy (10% the most frequent side effects). However, in our department we had previous successful experience in treating inguinal lymphocele with bleomycin without major complications and for that reason it was the agent of choice for this particular case. Elsandabesee et al. also reported a safe successful sclerotherapy with bleomycin in a recurrent massive inguinal lymphocele [56].

## 4. Conclusion

As a potential severe complication following radical pelvic lymphadenectomy, symptomatic lymphoceles should be promptly treated. If daily high volume drainage persists after percutaneous catheter drainage alone, sclerotherapy should be performed. In the absence of robust scientific evidence and expert consensus for the safest and most effective sclerosing agent, individual experience at the management of such situation should guide our options. There is a higher experience with the use of bleomycin in inguinal lymphoceles than in abdominopelvic ones. This case reports adds empirical evidence and reinforces the use of bleomycin as a safe and excellent sclerosing agent. It should be regard as a first-line agent in refractory abdominopelvic lymphocele.

If it is true that there is no matter of debate regarding the first-line treatment of significant, symptomatic, or complicated lymphoceles by percutaneous techniques through interventional radiology with combined catheter drainage and transcatheter sclerotherapy, the medical community cannot reach a consensual sclerosing agent. Search for the most effective and efficient sclerosing agent is still a continuing process and requires prospective, well-designed randomized studies with comparison of different agents. The present case contributes to the growing experience in this particular matter.

## Figures and Tables

**Table 1:** Studies in treatment modalities for pelvic lymphoceles.

References	*N*	Treatment modality	Sclerosing agent	Success rate	Side effects/complications
Surgical treatment					
Radosa et al., 2013 [27]	132	Laparoscopic drainage	NA	93%	Intraoperative 9.8% Postoperative 5.9%
Khoder et al., 2012 [28]	105	Laparoscopic drainage	NA	97%	Not mentioned/unknown
Hsu et al., 2000 [57]	81	Laparoscopic drainage	NA	94%	Intraoperative (5%): (i) laryngospasm (*n* = 1) (ii) inferior epigastric artery injury (*n* = 1) (iii) mild renal capsule hematoma (*n* = 1) (iv) bladder injury (*n* = 1) Postoperative (4%): (i) trocar site hernia (*n* = 1) (ii) urinary retention (*n* = 2)
Cadrobbi et al., 1999 [58]	12	Laparoscopic drainage	NA	92%	8%: bleeding from the peritoneal window (*n* = 1)
Ostrowski et al., 2000 [59]	9	Laparoscopic drainage	NA	89%	None
Melvin et al., 1997 [60]	8	Laparoscopic drainage	NA	75% (only 6 follow-up)	None
Gill et al., 1995 [61]	38	Open drainage (*n* = 26) versus laparoscopic drainage (*n* = 12)	NA	73% versus 100%	Open drainage (12%): (i) prolonged ileus (*n* = 1) (ii) transection of the ureter of a transplant kidney (*n* = 1) (iii) pneumonitis (*n* = 1) Laparoscopic drainage (8%): cystotomy requiring open repair (*n* = 1)

Minimal invasive therapy					
Kim et al., 1999 [30]	23	Percutaneous catheter drainage	NA	96%∗ to 100%∗∗	17%: (i) secondary infection (*n* = 1) (ii) skin infection (*n* = 2) (iii) catheter dislodgement (*n* = 1)
Kurata et al., 2003 [31]	10	Percutaneous catheter drainage	NA	90%	None
Conte et al., 1990 [32]	8	Percutaneous catheter drainage	NA	88% to 100%∗∗	None
White et al., 1985 [29]	11	Simple aspiration (*n* = 2) and percutaneous catheter drainage (*n* = 9)	NA	82%	27% lymphocele infection (*n* = 3)
Jensen et al., 1986 [33]	8	Simple aspiration (*n* = 7) and percutaneous catheter drainage (*n* = 1)	NA	75% (only 7 follow-up)	None
Akhan et al., 2007 [34]	60	Percutaneous catheter drainage (*n* = 10) and sclerotherapy (*n* = 50)	Ethanol	70% and 98%∗	12% (i) catheter dislodgement (*n* = 1) (ii) secondary infection (*n* = 6)
Akhan et al., 2000 [35]	38	Percutaneous catheter drainage (*n* = 7) and sclerotherapy (*n* = 31)	Ethanol	97% to 100%∗∗	Not mentioned/unknown
Zuckerman and Yeager, 1997 [36]	32	Sclerotherapy	Ethanol	94%	9%: secondary infection (*n* = 3)
Sawhney et al., 1996 [37]	14	Sclerotherapy	Ethanol	93% to 100%∗∗	None
Akhan et al., 1992 [38]	8	Sclerotherapy	Ethanol	88% to 100%∗∗	None
Kuzuhara et al., 1994 [39]	4	Sclerotherapy	Ethanol	75%	Not mentioned/unknown
Alago et al., 2013 [40]	70	Percutaneous catheter drainage (*n* = 52) versus sclerotherapy (*n* = 18)	Povidone-iodine	74% to 97%∗∗ versus 100%∗	34% (i) pericatheter fluid leakage (*n* = 4) (ii) catheter dislodgement (*n* = 3) (iii) catheter occlusion (*n* = 9) (iv) secondary infection of the collection (*n* = 4)
Rivera et al., 1996 [41]	19	Sclerotherapy	Povidone-iodine	62.5% to 100%∗∗	15%: secondary infection (*n* = 3)
Montalvo et al., 1996 [42]	17	Sclerotherapy	Povidone-iodine	82% to 100%∗∗	Not mentioned/unknown
Cohan et al., 1988 [43]	13	Percutaneous catheter drainage (*n* = 6) and sclerotherapy (*n* = 7)	Povidone-iodine	83%∗ 86%	None
Gilliland et al., 1989 [44]	9	Sclerotherapy (*n* = 9)	Povidone-iodine	89%	None
Shokeir et al., 1993 [45]	30	Percutaneous catheter drainage (*n* = 9) and sclerotherapy (*n* = 21)	Tetracycline	93%	Not mentioned/unknown
Caliendo et al., 2001 [46]	21	Sclerotherapy	Doxycycline	95%∗	27%: minor discomfort or transient mild temperature elevation (*n* = 5)
Folk and Musa, 1995 [47]	3	Sclerotherapy	Doxycycline	67%	None
VanSonnenberg et al., 1986 [48]	14	Percutaneous catheter drainage (*n* = 10) and sclerotherapy (*n* = 4)	Tetracycline povidone-iodine	79%	Not mentioned/unknown
Mahrer et al., 2010 [4]	43	Sclerotherapy	Povidone-iodine ethanol doxycycline	77%∗	35%: (i) cellulitis (*n* = 1) (ii) severe testicular pain (*n* = 1) (iii) ureteral obstruction and increased creatinine level (*n* = 1) (iv) acute tubular necrosis (*n* = 1) (v) localized peritonitis (*n* = 11)
Chin et al., 2003 [49]	8	Sclerotherapy	Fibrin glue	75%∗	38%: (i) clogging of drainage catheters (*n* = 2) (ii) catheter dislodgement (*n* = 1)
Kerlan et al., 1997 [50]	4	Sclerotherapy	Bleomycin	100%∗	25% transient episode of fever (*n* = 1)

Several modalities					
Atray et al., 2004 [62]	36	Simple aspiration (*n* = 4), percutaneous catheter drainage (*n* = 12), sclerotherapy (*n* = 4), open drainage (*n* = 8), and laparoscopic drainage (*n* = 8)	Ethanol	100% 67% 75% 88% 88%	Sclerotherapy: infection (*n* = 3) Open drainage infection (*n* = 1)
Ziȩtek et al., 2007 [63]	14	Percutaneous catheter drainage (*n* = 14) followed by laparoscopic drainage (*n* = 7)		50% 86%	None

*N*: number of lymphoceles. NA: not applied. ∗After multiple sessions. ∗∗Considering asymptomatic recurrence.

## References

[1] Kobayashi T, Inoue S (1950). Pelvic lymphocyst. *Clinical Obstetrics and Gynecology*.

[2] Kim HY, Kim JW, Kim SH, Kim YT, Kim JH (2004). An analysis of the risk factors and management of lymphocele after pelvic lymphadenectomy in patients with gynecologic malignancies. *Cancer Treatment and Research*.

[3] Karcaaltincaba M, Akhan O (2005). Radiologic imaging and percutaneous treatment of pelvic lymphocele. *European Journal of Radiology*.

[4] Mahrer A, Ramchandani P, Trerotola SO, Shlansky-Goldberg RD, Itkin M (2010). Sclerotherapy in the management of postoperative lymphocele. *Journal of Vascular and Interventional Radiology*.

[5] Dodd GD, Rutledge F, Wallace S (1970). Postoperative pelvic lymphocysts. *The American Journal of Roentgenology, Radium Therapy, and Nuclear Medicine*.

[6] Braun WE, Banowsky LH, Straffon RA (1974). Lymphoceles associated with renal transplantation. Report of 15 cases and review of the literature. *The American Journal of Medicine*.

[7] Glass LL, Cockett ATK (1998). Lymphoceles: diagnosis and management in urologic patients. *Urology*.

[8] Jensen SR, Voegeli DR, McDermott JC, Crummy AB, Turnipseed WD (1986). Lymphatic disruption following abdominal aortic surgery. *Cardiovascular and Interventional Radiology*.

[9] Levi ADO (1999). Treatment of a retroperitoneal lymphocele after lumbar fusion surgery with intralesional povidone iodine: technical case report. *Neurosurgery*.

[10] Leitner DW, Sherwood RC (1983). Inguinal lymphocele as a complication of thighplasty. *Plastic and Reconstructive Surgery*.

[11] Greenberg BM, Perloff LJ, Grossman RA, Naji A, Barker CF (1985). Treatment of lymphocele in renal allograft recipients. *Archives of Surgery*.

[12] Tam KF, Lam KW, Chan KK, Ngan HYS (2008). Natural history of pelvic lymphocysts as observed by ultrasonography after bilateral pelvic lymphadenectomy. *Ultrasound in Obstetrics and Gynecology*.

[13] Achouri A, Huchon C, Bats AS, Bensaid C, Nos C, Lécuru F (2013). Complications of lymphadenectomy for gynecologic cancer. *European Journal of Surgical Oncology*.

[14] Gauthier T, Uzan C, Lefeuvre D (2012). Lymphocele and ovarian cancer: risk factors and impact on survival. *Oncologist*.

[15] Metcalf KS, Peel KR (1993). Lymphocele. *Annals of the Royal College of Surgeons of England*.

[16] Achouri A, Huchon C, Bats A, Bensaïd C, Nos C, Lécuru F (2012). Postoperative lymphocysts after lymphadenectomy for gynaecological malignancies: preventive techniques and prospects. *European Journal of Obstetrics Gynecology and Reproductive Biology*.

[17] Morice P, Lassau N, Pautier P, Haie-Meder C, Lhomme C, Castaigne D (2001). Retroperitoneal drainage after complete para-aortic lymphadenectomy for gynecologic cancer: a randomized trial. *Obstetrics and Gynecology*.

[18] Clarke-Pearson DL, Jelovsek FR, Creasman WT (1983). Thromboembolism complicating surgery for cervical and uterine malignancy: incidence, risk factors, and prophylaxis. *Obstetrics and Gynecology*.

[19] Kropfl D, Krause R, Hartung R, Pfeiffer R, Behrendt H (1987). Subcutaneous heparin injection in the upper arm as a method of avoiding lymphoceles after lymphadenectomies in the lower part of the body. *Urologia Internationalis*.

[20] Ghezzi F, Uccella S, Cromi A (2012). Lymphoceles, lymphorrhea, and lymphedema after laparoscopic and open endometrial cancer staging. *Annals of Surgical Oncology*.

[21] Petru E, Tamussino K, Lahousen M, Winter R, Pickel H, Haas J (1989). Pelvic and paraaortic lymphocysts after radical surgery because of cervical and ovarian cancer. *American Journal of Obstetrics and Gynecology*.

[22] Abu-Rustum NR, Alektiar K, Iasonos A (2006). The incidence of symptomatic lower-extremity lymphedema following treatment of uterine corpus malignancies: a 12-year experience at Memorial Sloan-Kettering Cancer Center. *Gynecologic Oncology*.

[23] Franchi M, Trimbos JB, Zanaboni F (2007). Randomised trial of drains versus no drains following radical hysterectomy and pelvic lymph node dissection: a European Organisation for Research and Treatment of Cancer-Gynaecological Cancer Group (EORTC-GCG) study in 234 patients. *European Journal of Cancer*.

[24] Charoenkwan K, Kietpeerakool C (2010). Retroperitoneal drainage versus no drainage after pelvic lymphadenectomy for the prevention of lymphocyst formation in patients with gynaecological malignancies. *The Cochrane Library*.

[25] Olsson CA, Willscher MK, Filoso AM, Cho SI (1976). Treatment of posttransplant lymphoceles: internal versus external drainage. *Transplantation Proceedings*.

[26] McCullough CS, Soper NJ, Clayman RV, So SS, Jendrisak MD, Hanto DW (1991). Laparoscopic drainage of a posttransplant lymphocele. *Transplantation*.

[27] Radosa MP, Diebolder H, Camara O, Mothes A, Anschuetz J, Runnebaum IB (2013). Laparoscopic lymphocele fenestration in gynaecological cancer patients after retroperitoneal lymph node dissection as a first-line treatment option. *BJOG: An International Journal of Obstetrics and Gynaecology*.

[28] Khoder WY, Gratzke C, Haseke N, Herlemann A, Stief CG, Becker AJ (2012). Laparoscopic marsupialisation of pelvic lymphoceles in different anatomic locations following radical prostatectomy. *European Urology*.

[29] White M, Mueller PR, Ferrucci JT (1985). Percutaneous drainage of postoperative abdominal and pelvic lymphoceles. *American Journal of Roentgenology*.

[30] Kim J, Jeong Y, Kim Y, Kang H, Choi H (1999). Postoperative pelvic lymphocele: treatment with simple percutaneous catheter drainage. *Radiology*.

[31] Kurata H, Aoki Y, Tanaka K (2003). Simple one-step catheter placement for the treatment of infected lymphocele. *European Journal of Obstetrics Gynecology and Reproductive Biology*.

[32] Conte M, Panici PB, Guariglia L, Scambia G, Greggi S, Mancuso S (1990). Pelvic lymphocele following radical para-aortic and pelvic lymphadenectomy for cervical carcinoma: Incidence rate and percutaneous management. *Obstetrics and Gynecology*.

[33] Jensen SR, Voegeli DR, McDermott JC, Crummy AB (1986). Percutaneous management of lymphatic fluid collections. *CardioVascular and Interventional Radiology*.

[34] Akhan O, Karcaaltincaba M, Ozmen MN, Akinci D, Karcaaltincaba D, Ayhan A (2007). Percutaneous transcatheter ethanol sclerotherapy and catheter drainage of postoperative pelvic lymphoceles. *CardioVascular and Interventional Radiology*.

[35] Akhan O, Ozmen M, Karcaaltincaba M (2000). Percutaneous transcatheter ethanol sclerotherapy postoperative pelvic lymphoceles. *Cardiovascular and Interventional Radiology*.

[36] Zuckerman DA, Yeager TD (1997). Percutaneous ethanol sclerotherapy of postoperative lymphoceles. *American Journal of Roentgenology*.

[37] Sawhney R, D'Agostino HB, Zinck S (1996). Treatment of postoperative lymphoceles with percutaneous drainage and alcohol sclerotherapy. *Journal of Vascular and Interventional Radiology*.

[38] Akhan O, Cekirge S, Ozmen M, Besim A (1992). Percutaneous transcatheter ethanol sclerotherapy of postoperative pelvic lymphoceles. *CardioVascular and Interventional Radiology*.

[39] Kuzuhara K, Nishimori S, Kurooka Y (1994). Conservative treatment of lymphocele after renal transplantation, using 95% ethanol instillation. *Transplantation Proceedings*.

[40] Alago W, Deodhar A, Michell H (2013). Management of postoperative lymphoceles after lymphadenectomy: percutaneous catheter drainage with and without povidone-iodine sclerotherapy. *CardioVascular and Interventional Radiology*.

[41] Rivera M, Marcén R, Burgos J (1996). Treatment of posttransplant lymphocele with povidone-iodine sclerosis: long-term follow-up. *Nephron*.

[42] Montalvo BM, Yrizarry JM, Javier Casillas V (1996). Percutaneous sclerotherapy of lymphoceles related to renal transplantation. *Journal of Vascular and Interventional Radiology*.

[43] Cohan RH, Saeed M, Schwab SJ, Perlmutt LM, Dunnick NR (1988). Povidone-iodine sclerosis of pelvic lymphoceles: a prospective study. *Urologic Radiology*.

[44] Gilliland JD, Spies JB, Brown SB, Yrizarry JM, Greenwood LH (1989). Lymphoceles: percutaneous treatment with povidone-iodine sclerosis. *Radiology*.

[45] Shokeir AA, El-Diasty TA, Ghoneim MA (1993). Percutaneous treatment of lymphocele in renal transplant recipients. *Journal of Endourology*.

[46] Caliendo MV, Lee DE, Queiroz R, Waldman DL (2001). Sclerotherapy with use of doxycycline after percutaneous drainage of postoperative lymphoceles. *Journal of Vascular and Interventional Radiology*.

[47] Folk JJ, Musa AG (1995). Management of persistent lymphocele by sclerotherapy with doxycycline. *European Journal of Obstetrics Gynecology and Reproductive Biology*.

[48] VanSonnenberg E, Wittich GR, Casola G (1986). Lymphoceles: imaging characteristics and percutaneous management. *Radiology*.

[49] Chin AI, Ragavendra N, Hilborne L, Gritsch HA (2003). Fibrin sealant sclerotherapy for treatment of lymphoceles following renal transplantation. *Journal of Urology*.

[50] Kerlan RK, LaBerge JM, Gordon RL, Ring EJ (1997). Bleomycin sclerosis of pelvic lymphoceles. *Journal of Vascular and Interventional Radiology*.

[51] Nikbakhsh N, Amiri AP, Hoseinzadeh D (2011). Bleomycin in the treatment of 50 cases with malignant pleural effusion. *Caspian Journal of Internal Medicine*.

[52] Tan C, Sedrakyan A, Browne J, Swift S, Treasure T (2006). The evidence on the effectiveness of management for malignant pleural effusion: a systematic review. *European Journal of Cardio-Thoracic Surgery*.

[53] Cuervo JL, Galli E, Eisele G (2011). Lymphatic malformations: percutaneus treatment with bleomycin. *Archivos Argentinos de Pediatria*.

[54] Rozman Z, Thambidorai CR, Zaleha AM, Zakaria Z, Zulfiqar MA (2011). Lymphangioma: is intralesional bleomycin sclerotherapy effective?. *Biomedical Imaging and Intervention Journal*.

[55] Khorram O, Stern JL (1993). Bleomycin sclerotherapy of an intractable inguinal lymphocyst. *Gynecologic Oncology*.

[56] Elsandabesee D, Sharma B, Preston J, Ostrowski J, Nieto J (2004). Sclerotherapy with bleomycin for recurrent massive inguinal lymphoceles following partial vulvectomy and bilateral lymphadenectomy—case report and literature review. *Gynecologic Oncology*.

[57] Hsu TH, Gill IS, Grune MT (2000). Laparoscopic lymphocelectomy: a multi-institutional analysis. *Journal of Urology*.

[58] Cadrobbi R, Zaninotto G, Rigotti P, Baldan N, Sarzo G, Ancona E (1999). Laparoscopic treatment of lymphocele after kidney transplantation. *Surgical Endoscopy*.

[59] Ostrowski M, Lubikowski J, Kowalczyk M, Power J (2000). Laparoscopic lymphocele drainage after renal transplantation. *Annals of Transplantation*.

[60] Melvin WS, Bumgardner GL, Davies EA, Elkhammas EA, Henry ML, Ferguson RM (1997). The laparoscopic management of post-transplant lymphocele: a critical review. *Surgical Endoscopy*.

[61] Gill IS, Hodge EE, Munch LC (1995). Transperitoneal marsupialization of lymphoceles: a comparison of laparoscopic and open techniques. *Journal of Urology*.

[62] Atray NK, Moore F, Zaman F (2004). Post transplant lymphocele: a single centre experience. *Clinical Transplantation*.

[63] Ziȩtek Z, Sulikowski T, Tejchman K (2007). Lymphocele after kidney transplantation. *Transplantation Proceedings*.

